# Fostering Adequate Data Reporting of Fourier Transform Ion Cyclotron Resonance Mass Spectrometry-Based Environmental Studies

**DOI:** 10.1021/acsestwater.4c00431

**Published:** 2024-06-11

**Authors:** Xiaohan Mo, Penghui Du, T.-W. Dominic Chan, Pang Chui Shaw, Alex Tat-Shing Chow

**Affiliations:** † Earth and Environmental Sciences Programme, The Chinese University of Hong Kong, Shatin 999077, Hong Kong SAR, China; ‡ Department of Chemistry, The Chinese University of Hong Kong, Shatin 999077, Hong Kong SAR, China; ⊥ School of Life Sciences, The Chinese University of Hong Kong, Shatin 999077, Hong Kong SAR, China; ¶ Li Dak Sum Yip Yio Chin R&D Centre for Chinese Medicine, The Chinese University of Hong Kong, Shatin 999077, Hong Kong SAR, China

Fourier transform ion cyclotron resonance mass spectrometry (FT-ICR MS) is a state-of-the-art ultra-high-resolution mass spectrometry method. Its exceptional resolving power has greatly expanded our analytical ability to discern, identify, and quantify diverse molecular compounds in complex systems, such as natural organic matter (NOM).^1^ Over the past two decades, interest in using FT-ICR MS for environmental studies has grown rapidly. With “FT-ICR MS” and “organic matter” as keywords, there were more than 1000 publications in Web of Science, with >60% published in first-quartile journals such as *Environmental Science & Technology* and *ACS ES&T Water*. More than half of the relevant articles were published in the past two years, and the number of relevant studies is expected to increase exponentially. As the FT-ICR MS community expands, we note with concern that the inadequate data reporting in some recent environmental studies may limit the discussions, the scientific confidence, and the potential for these FT-ICR MS data sets to be reused. On the basis of a comprehensive investigation of relevant studies, we discussed some of the most typical and common concerns that should be better addressed in future studies.

## Account for Spatial and Temporal Variability

Field samples are known to be highly complex and heterogeneous. As such, unbiased sample collection representing the desired field conditions is critical for a solid environmental study.^1−3^ The description of field experiments should present exactly what, where, and how authors conducted, including but not limited to, sampling design, field conditions, storage, and extraction of analytes.^1^ The justification of the choice of the sampling points and time should also be clearly explained. For example, considering water sampling from a dynamic water body such as a river or an estuary, essential details should include the exact time and location coordinates for each sample collection. Samples collected before or after a heavy rainstorm, incoming or outgoing tide, and upstream or downstream of river confluence could represent NOM from different sources and/or biogeochemical processes. Furthermore, the sampling depth (e.g., surface water or near the bottom of the stream), location (e.g., at the edge or the middle of the river), and other environmental conditions (e.g., stagnant or flowing water) should be described. There are great spatial and temporal variations in the quantity and quality of NOM. Unfortunately, many recent articles failed to report this essential information.

## Detailed Sample Preparation for Reproductivity

For many scientific studies to characterize NOM in environmental samples, it is typical and reasonable that sample replication (e.g., *n* ≥ 3) is designed for general analytical techniques (e.g., total organic carbon quantification and ultraviolet–visible spectroscopy) to ensure the quality assurance and control of the measurements. However, given the fact of the high testing fee and limited accessibility of FT-ICR MS worldwide, many researchers often sacrifice sample replication or use composite samples to conserve costs and resources. Therefore, using representative environmental samples with an accurate sample preparatory procedure must be addressed; if not, misinterpretations can be raised. In addition to the time and location discussed above, the detailed sampling protocol, including the number of replications, volume or mass used in composite samples (if any), sampling device used, types of containers, duration of storage, materials of filtration membrane and solid phase extraction, glassware cleaning procedures, and the special handling procedure to avoid potential contamination or degradation during storage, should be carefully planned and performed. FT-ICR MS is a highly sensitive analytical technique such that a minor discrepancy could impact the composition of NOM in samples and consequently, the results of the analysis.^3−5^ In addition, reporting blank and certified reference controls and replicate injections of 10–20% of the samples have been recommended for quality assurance and control.^2^ Such information is, however, often inadvertently neglected in research articles.

## Specific Information about the Instrument Is Required

**Figure 1:**
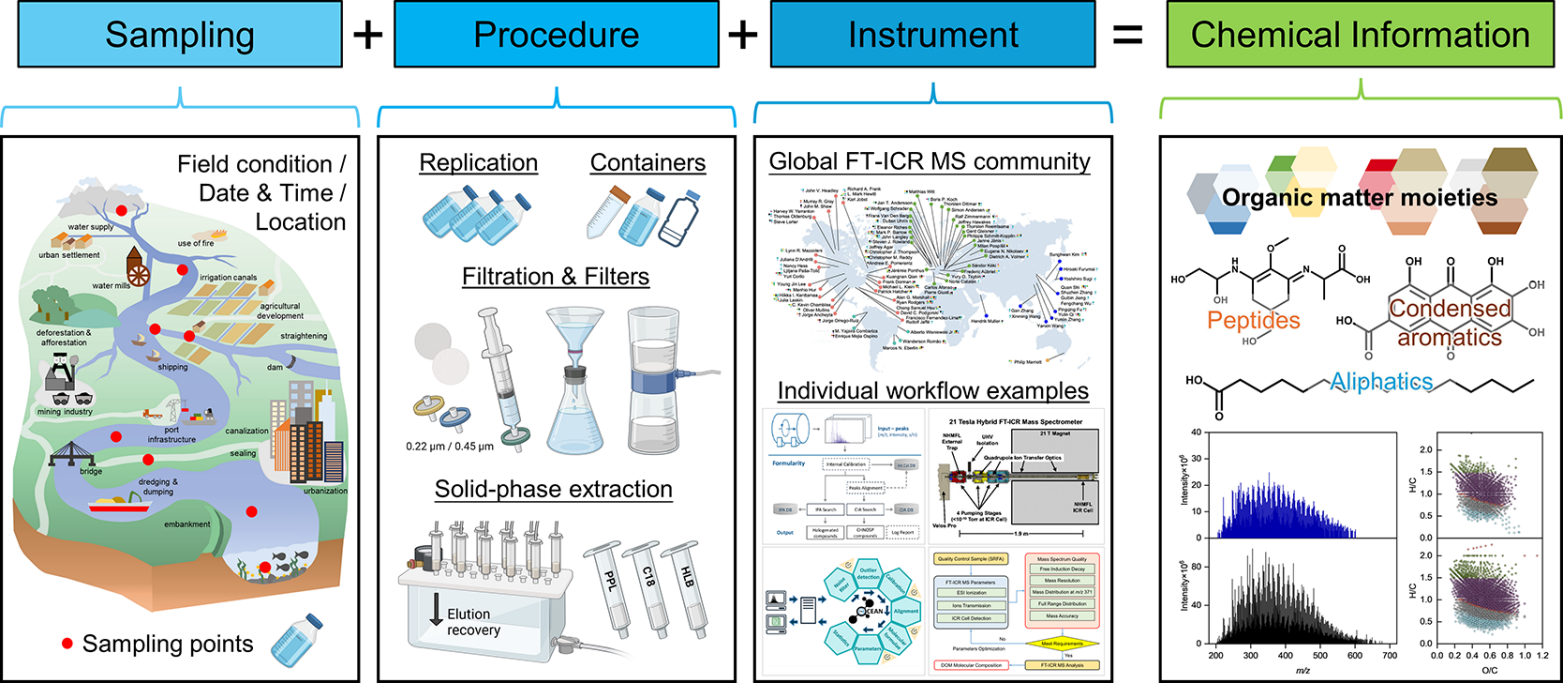
Detailing the sample collection, preparation procedures, and operating laboratories can provide more opportunities for sample comparisons across spatial and temporal scales. Illustrations for “sampling” are reprinted with permission under a Creative Commons Attribution 4.0 International License from ref (9). Copyright 2021 Springer Nature. Illustrations for the “Global FT-ICR MS community” are reprinted with permission under a Creative Commons Attribution 4.0 International License from ref (10). Copyright 2022 Springer Nature. Illustrations for “Individual workflow examples” are reprinted with permission from ref (11) (Copyright 2017 American Chemical Society), ref (12) (Copyright 2015 American Chemical Society), ref (13) (Copyright 2020 American Chemical Society), and ref (14) (Copyright 2020 American Chemical Society).

Reporting only the model of the instrument (e.g., 9.4 T Apex Ultra FT-ICR, 12 T SolariX FT-ICR) is insufficient for data presentation and interpretation. Despite its wide use in NOM research, FT-ICR MS is still not commonly available to scientific communities. Most researchers ship their samples to certain laboratories or user facilities equipped with FT-ICR MS. Results, typically expressed with formula assignment, are delivered back to the users. Because of this mode of operation, researchers generally cannot manipulate any parameters of the instruments that are set by the laboratories and have limited information about calibration and other essential data quality assurance procedures. In fact, each FT-ICR MS laboratory has developed individual workflows for optimized performance (Figure 1), including but not limited to instrument settings, detection limits, and injection solvents.^2^ The magnetic field strength, ionization efficiency, ICR cell, ion transfer optics, and different interpretation strategies (e.g., noise peaks, calibration, and formula assignment) can affect the results.^6,7^ Interlaboratory comparison studies found that the assigned molecular formula lists acquired from different instruments appear distinct even for the same samples.^2,8^ Direct comparisons of FT-ICR MS data sets among different instruments remain a great challenge.^2^ It is therefore essential to specify which FT-ICR MS laboratory is used so that the community can refer to technical details provided by the operating laboratories. A rough estimate shows that approximately one-third of the studies in the past year failed to report where they performed the FT-ICR MS analysis. Disclosing these details will significantly contribute to the quality of publications and bolster scientific confidence in the research findings. Noticeably, these considerations may not be applied to NOM characterization using Orbitrap mass spectrometry because researchers generally can have access and control over their own instruments. Researchers are responsible for the instruments’ performance and can report the operating parameters and conditions in the Supporting Information.

Previous publications have highlighted several challenges and considerations for interlaboratory comparison with FT-ICR MS data.^7,8^ In addition, we aim to remind the expanding community in a timely manner that adequate data reporting is as important as the technology itself. Detailing the sample collection, preparation procedures, and operating laboratories is a simple yet critical step that can provide more opportunities for sample comparisons across spatial and temporal scales (Figure 1). We hope that each data set generated by FT-ICR MS can be valuable for the community, reusable for further meta-analyses, and able to advance our understanding of the changing environment.
